# Deep learning for the prediction of clinical outcomes in internet-delivered CBT for depression and anxiety

**DOI:** 10.1371/journal.pone.0272685

**Published:** 2023-11-27

**Authors:** Niranjani Prasad, Isabel Chien, Tim Regan, Angel Enrique, Jorge Palacios, Dessie Keegan, Usman Munir, Ryutaro Tanno, Hannah Richardson, Aditya Nori, Derek Richards, Gavin Doherty, Danielle Belgrave, Anja Thieme

**Affiliations:** 1 Microsoft Health Futures, Microsoft Research, Cambridge, United Kingdom; 2 Cambridge University, Cambridge, United Kingdom; 3 Cambridge Respiratory Innovations, Cambridge, United Kingdom; 4 SilverCloud Science, SilverCloud Health, Dublin, Ireland; 5 E-Mental Health Group, School of Psychology, Trinity College Dublin, Dublin, Ireland; 6 GSK, London, United Kingdom; 7 School of Computer Science and Statistics, Trinity College Dublin, Dublin, Ireland; Public Library of Science, UNITED KINGDOM

## Abstract

In treating depression and anxiety, just over half of all clients respond. Monitoring and obtaining early client feedback can allow for rapidly adapted treatment delivery and improve outcomes. This study seeks to develop a state-of-the-art deep-learning framework for predicting clinical outcomes in internet-delivered Cognitive Behavioural Therapy (iCBT) by leveraging large-scale, high-dimensional time-series data of client-reported mental health symptoms and platform interaction data. We use de-identified data from 45,876 clients on SilverCloud Health, a digital platform for the psychological treatment of depression and anxiety. We train deep recurrent neural network (RNN) models to predict whether a client will show reliable improvement by the end of treatment using clinical measures, interaction data with the iCBT program, or both. Outcomes are based on total improvement in symptoms of depression (Patient Health Questionnaire-9, PHQ-9) and anxiety (Generalized Anxiety Disorder-7, GAD-7), as reported within the iCBT program. Using internal and external datasets, we compare the proposed models against several benchmarks and rigorously evaluate them according to their predictive accuracy, sensitivity, specificity and AUROC over treatment. Our proposed RNN models consistently predict reliable improvement in PHQ-9 and GAD-7, using past clinical measures alone, with above 87% accuracy and 0.89 AUROC after three or more review periods, outperforming all benchmark models. Additional evaluations demonstrate the robustness of the achieved models across (i) different health services; (ii) geographic locations; (iii) iCBT programs, and (iv) client severity subgroups. Results demonstrate the robust performance of dynamic prediction models that can yield clinically helpful prognostic information ready for implementation within iCBT systems to support timely decision-making and treatment adjustments by iCBT clinical supporters towards improved client outcomes.

## Introduction

Depression and anxiety are primary drivers of disability worldwide [1]. Treatment via cognitive behavioural therapy (CBT) has been established through decades of research, and in recent years, the active ingredients in CBT have been disseminated through the internet and proven effective in yielding reliable improvement in clinical symptoms [2–5]. While internet-based CBT (iCBT) offers many unique advantages, in particular flexible access to treatment resources, maintaining user engagement with digitally delivered behavioural interventions remains challenging [6]. The involvement of a clinical supporter, whose role is to encourage and facilitate clients’ use of the digital intervention, has been shown to lead to better treatment engagement and outcomes than self-guided therapy [7, 8]. These clinical supporters frequently communicate with their clients via online messages or telephone conversations to facilitate their learning and the application of self-taught mental health management techniques. The specific skills and techniques of CBT treatment, alongside the clinical relationship, seek to address cognitive and behavioral processes to drive change for clients. The acquisition and practical application of these skills by clients is an essential outcome of all CBT-based treatments [9, 10].

Using technology allows supporters to gain information about clients’ engagement with the treatment and potentially to intervene based on this information. This is commensurate with the Feedback-Informed Treatment (FIT) paradigm [11] that builds on the routine outcome monitoring (ROM) framework, as continuous monitoring of symptoms during treatment is critical for effective clinical decision-making [12]. Empirical evidence demonstrates that therapists use of FIT reduces the likelihood of patient deterioration and can lead to lower average duration and cost of treatment compared to controls [13–16]. Indeed, guidelines recommend that depression and anxiety treatments should follow through to remission for clients, yet only about 50% of clients reach remission. Feedback allows the supporting clinician to understand if the treatment is progressing as expected, to understand early if the client is benefitting or likely to benefit, and to make any necessary adjustments to improve the likelihood of treatment response. FIT is independent of any one theoretical approach and focuses on the importance of a culture of feedback throughout therapy. In tandem, a focus on deliberate action to improve quality and outcomes has proved salient [11].

In the case of depression and anxiety, just over half of all clients are expected to respond to treatment [17–20]. Insights early in treatment about prospective outcomes can enable clinical supporters of iCBT interventions to engage in more timely and proactive intervention by increasing their level of client support or re-assessing the suitability of the chosen treatment for an individual. These insights can aid decisions on whether a client needs to be stepped up to different care or whether more treatment sessions are needed for a particular client. This allows for more effective distribution of care resources as well as reductions in the negative impact of having a client remain too long on the wrong care pathway: literature has shown that (real-time) feedback to therapists on expected outcomes for a client can prevent symptom deterioration and improve treatment success [15, 21, 22].

Recent years have begun to see the development of machine learning models for risk stratification or prediction of outcomes from treatment where research is at an early stage when using only baseline data. Still, more sound evidence exists when using routine outcome monitoring data [23–25]. To date, within iCBT contexts, models have been built on modest and selective samples (ranging from fewer than 100 to ~2000 users) and have typically involved post-hoc analysis of RCT data or miscellaneous data sources, with mixed results [25–29]. While RCT data has the advantage that it is often less biased than data collected in a naturalistic cohort study, the modest sample sizes restrict the feasibility of high-capacity machine learning methods and limit the scope for robust validation.

In this work, we present the analysis of a large clinical sample of 45,876 mental health clients for predicting clinical outcomes. Our work leverages some unique opportunities afforded by iCBT interventions, which capture real-time data of client interactions with psychotherapy treatment alongside any changes in clinical symptoms over time. This scale of data also enables the application of a deep learning (DL) framework, which has been shown to achieve state-of-the-art performance in many applications with sequential data, including clinical time series [30]. DL methods can scale to large datasets compared to other statistical and machine learning methods. They have proven effective at modelling complex relationships from high-dimensional and unstructured data inputs—such as fine-grained platform interaction data—making them less reliant on significant feature engineering.

Our analysis aims to demonstrate the feasibility of developing robust classification models using DL for the early prediction of treatment outcomes. Furthermore, within the context of an iCBT intervention for the treatment of depression and anxiety, our research investigates: (i) what user data (in what combination) is most informative within such models, (ii) how early within treatment we can achieve predictions robust enough for use in clinical practice, and (iii) how well do the best-performing models generalize to other data populations.

## Materials and methods

### Data source and study population

This study leverages de-identified clinical measures and iCBT program interaction data from SilverCloud Health. This evidence-based, online self-administered platform delivers low-intensity iCBT alongside feedback from trained clinical supporters. Within the UK, SilverCloud services are predominantly accessed via client referrals from their GP or other healthcare professionals to IAPT (Improving Access to Psychological Therapies), a program that offers talking therapies to adults to help overcome depression and anxiety. IAPT offers treatment within a stepped care service model that ensures that the most effective but least resource-intensive treatment is delivered first, and care is only stepped-up to more intensive face-to-face treatments if required [31]. In this model, clients initially tend to access self-help oriented treatments, which are referred to as ‘low intensity’ (LI) interventions and that are usually delivered by clinical supporters [17]. SilverCloud Health is the most accessed online LI intervention. It is available in over 80% of IAPT services and offers over 35 treatment programs [32, 33].

Amongst the most popular and most accessed is the “Space from Depression and Anxiety” program. For our data-intense analysis, we considered all clients enrolled in this program between January 2015 and March 2019. The program consists of eight core modules covering fundamental CBT principles for treating symptoms of depression and anxiety. Content is delivered using textual and audio-visual materials, interactive tools (journals, quizzes, or mood trackers) and personal stories. Clients receive access to the iCBT program for up to 6 months; however, the core treatment is centred around 8 review periods (at intervals of 1 to 2 weeks), during which clinical supporters guide clients. These clinical supporters are a specially trained cohort of Psychological Wellbeing Practitioners (PWPs, https://www.instituteforapprenticeships.org/apprenticeship-standards/psychological-wellbeing-practitioner), typically graduate psychologists, with further training in low-intensity CBT-based interventions, including iCBT. Their support involves reviewing the engagement their client achieves from week to week and writing feedback to the client on their work, aiming to provide person-centred care that ensures good client experience and desired clinical outcomes.

All users in this study consented (oral or written) for their de-identified data to be used in analyses for routine service evaluation and improvement. Matching the terms and conditions of the service, and user consent, permits the analysis of anonymous data for research purposes and improves the treatment platform’s effectiveness and service tools. Users were informed that this analysis might include profiling, machine learning or other techniques. Where individual-level data is used, additional safeguards such as anonymisation, use of pseudonyms (pseudonymisation) and limiting the set of individual data used (data minimization) are in place (SilverCloud Privacy notice: https://uk.silvercloudhealth.com/help/privacy/). Since this study does not obtain information through intervention or interaction with SilverCloud users and only uses secondary, fully de-identified data, it is not classified as human subjects research and, therefore, exempt from IRB review (Definition of Human Subjects Research: https://grants.nih.gov/policy/humansubjects/research.htm).

**Fig 1:**
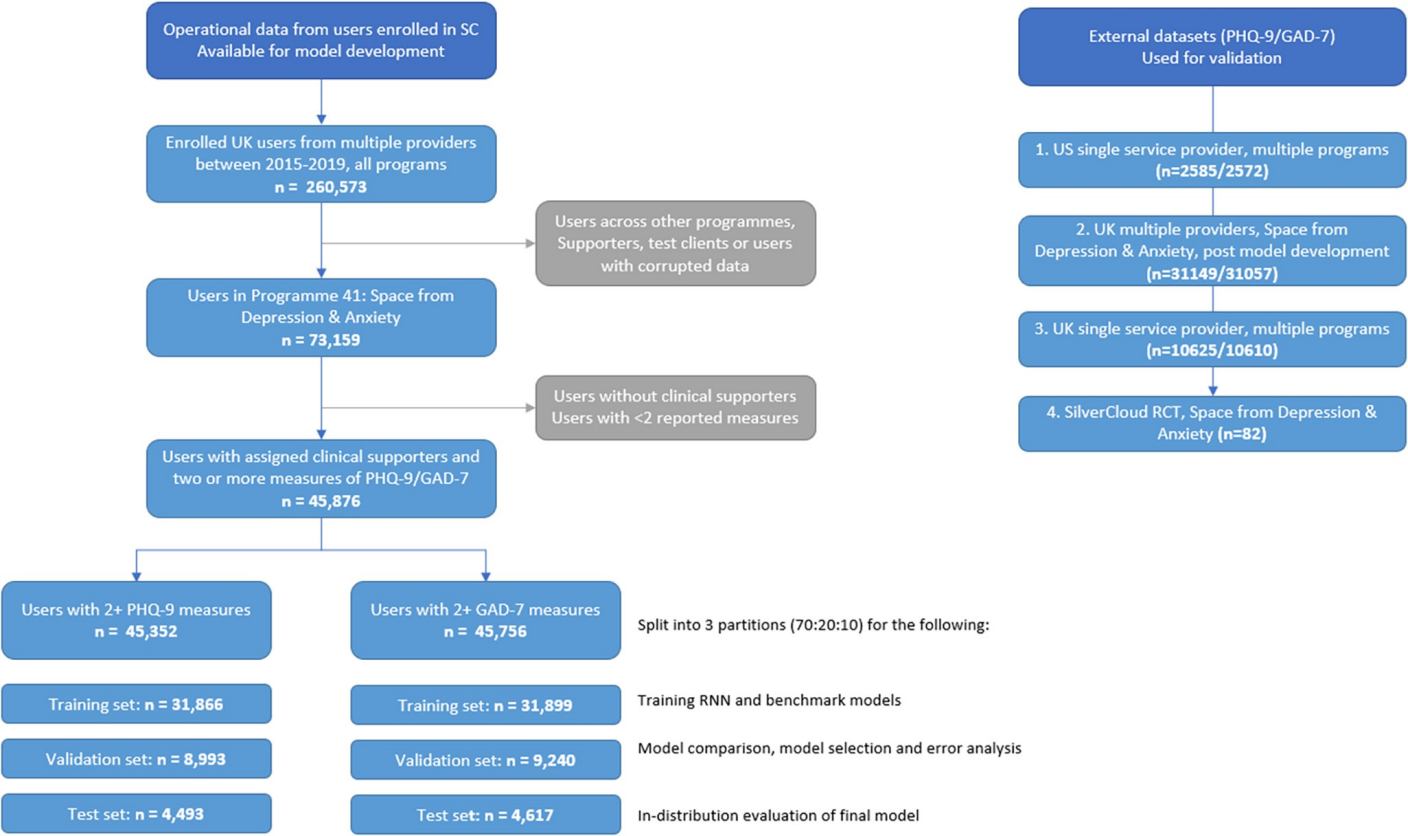
Overview of all datasets used in the analysis. Left: CONSORT diagram of data inclusion pipeline; right: Summary of external datasets (additional detail can be found in S4 File).

All steps taken to fully de-identify the data before any analysis are detailed in S1 File. This included the removal of free text entries, demographic information, and exact program dates. From this de-identified data, we selected clients who used the Space from Depression and Anxiety program, had an assigned clinical supporter, and completed clinical symptom measures at least twice. This resulted in 45,352 clients with regular reports of depression symptoms and 45,756 clients with regular reports of anxiety symptoms (Fig 1). In addition, to demonstrate the generalizability of our models, we also evaluated our models performance on 4 external datasets. These included one US (depression n = 2585, anxiety n = 2572) and two UK service providers (depression n = 41,774, anxiety = 41,667) as well as different iCBT programs (see S4 File for details). For one further, smaller UK single service program (n = 82 clients), we had demographic and clinical information for clients who participated in an RCT to test the effectiveness of SilverCloud Health. For all others, the nature of data access means that details on client diagnosis and other demographics such as their specific age, sex, race, ethnicity, or other socio-economic information were not available. Table 1 provides a structured overview of the key study elements following recent reporting guidelines for ML applications in clinical research [34, 35].

**Table 1:** Overview of the key study elements.

**Study design**	
Clinical question	Can we predict early and robustly reliable improvement (RI) from iCBT treatment for depression and anxiety?
Model task/output	Binary classification of treatment outcomes (RI by end of program) given access to clinical measurements, iCBT interaction data, or both.
Intended use of results/Target user	Predictions produced by the model can be made available to clinical supporters as part of management, to assist treatment adaptation for improved patient outcomes
**Study population + setting**	
Population	Clients receiving iCBT treatment for symptoms of depression and anxiety
Study setting	NHS IAPT services in the UK
Data source	SilverCloud Health Platform
Cohort selection (Exclusion/inclusion criteria)	Adult patients accessing the “Space from Depression and Anxiety Programme” with a clinical supporter. Client have at least 2 completed sets of PHQ-9 + GAD-7 measures within the enrolment period (96% of measures occur within the first 16 weeks)
**Patient demographics**	
Age	18+
Sex	Not provided
Race	Not provided
Ethnicity	Not provided
Socioeconomic status	Not provided
**Data sources**	
Data types	PHQ-9 and GAD-7 questionnaires; Interaction events with iCBT treatment
Data collection + transformation	See S1 File
Data structure + types	Ordinal (questionnaire scores); Count (interaction events)
Data partitions	See Fig 1
**Model architecture**	
ML methods & rationale	Recurrent neural networks (RNNs): can achieve high performances in modelling complex, high-dimensional data, and capturing long-term temporal dependencies in observations. Benchmarks: Logistic regression (LogR), Random forests (RF), Gradient boosting classifiers (GBMs), and Exponential moving averages (EMA)
Features	See Table 2
Data labels	Reliable improvement (see Table 3) between baseline (first) and last available (of a maximum of 8) self-reported measure for PHQ-9/GAD-7 respectively
Missingness	Last observation carried forward for label censoring due to user dropout before review 8
Hardware, software, packages	Model training + testing on the AzureML infrastructure, in Python 3.7 RNNs implemented via PyTorch
Data split	70:20:10 random split (training, validation and test)
Model training	RNN trained with cross-entropy loss for binary classification task. Hyperparameter tuning based on classification accuracy
Model parameters/ hyperparameters	3-layer RNN comprising 50-dim LSTM hidden layer + linear softmax. Dropout in LSTM layer with probability 0.4 Optimization using ADAM
**Model evaluation/ validation**	
Evaluation measures	Accuracy, AUROC, Sensitivity at fixed specificity
Internal model validation	Validation set (n = 9.2k) for model comparison and selection. Test set (n = 4.6k) for evaluation of final model
External model validation	Multiple external validation (see Table 6, S4 File)
Transparency, reproducibility, code reuse	Data is not available. Code is available on request from the corresponding author

Key study elements listed according to recent reporting guidelines by Hernandez-Boussard et al. [34] and Stevens et al. [35] for applications of machine learning in clinical research.

### Pre-processing and feature engineering

For all clients included in the analysis, the available data include: (i) *timestamped scores from the completion of clinical measures* assessing symptoms of depression and anxiety, as well as (ii) *timestamped entries for each client interaction with the iCBT program*. Together, these form the input features (i.e., “independent variables”) that are fed to our DL framework for predicting client outcomes (see Table 2).

**Table 2:** Component features for predicting reliable improvement.

Feature Type	# Dimensions	Description
Previous Total PHQ-9	1	Scale 0–27; based on PHQ-9 scores between week 0 (baseline) and 8 weeks for each client
Previous Total GAD-7	1	Scale 0–21; based on GAD-7 scores between week 0 (baseline) and 8 weeks for each client
Component PHQ-9 questions	9	Scale 0–3
Component GAD-7 questions	7	Scale 0–3
iCBT Program Interactions	1133	Counts of each unique type of program interaction as defined by their *action*, *section*, and *topic/tool ID*

The clinical measures comprise both the *individual component questions* and *total scores* for the nine-item Patient Health Questionnaire (PHQ-9) for depression (0–27 total score) [36] and the seven-item Generalized Anxiety Disorder scale (GAD-7) for anxiety (0–21 total score) [37]. These are extensively validated self-report instruments employed in routine outcome monitoring (ROM) within IAPT services. Clients are asked to complete these instruments by the clinical supporter, usually every 1–2 weeks during treatment, and ask the client to indicate: “Over the last two weeks, how often have you been bothered by any of the following problems?”. The PHQ-9 problem statements include: “Feeling down, depressed or hopeless”; and for the GAD-7: “Feeling nervous, anxious or on edge”. Each question is responded to on an ordinal scale from 0 (*not at all)* to 3 (*nearly every day*), with higher scores indicating more severe depression or anxiety symptoms.

We had the following information for the client’s interactions with the iCBT program. We have data on the **section** of the program that the client used, which can be *Content* pages that the person views; the use of interactive therapy *Tools*; their viewing or writing of a *Journal*; visiting their user *Profile* page; or whether they interacted with their supporter as part of the *Review* process. In addition to the program section, the data logs also include the types of **actions** taken concerning these sections. For example, the client can ‘view’, ‘bookmark’ or ‘complete’ treatment *Content*. For ‘*Content’* and ‘*Tools’* actions, the data also included a **topic id** and **tool id specifying the content or tool** accessed. The combination of section-action-topic/tool id taken together (e.g. {*Tools*, ‘add’, id: Thought-Feelings-Behavior cycle}) define a set of 1133 possible unique interactions that a client can take in the iCBT program. Thus, as our final feature set, we have the *count of each* of the 1133 unique iCBT program interactions (i.e., how often the client reviewed content, engaged with therapeutic tools, and so forth) that a client performed within a given review period. We explain what constitutes a review period next.

Since interactions with the iCBT program accumulate the more the client engages with treatment, and clinical measures are frequently completed over time, we needed to identify meaningful time intervals to assess or predict the client’s progress. We chose *review periods* as our prediction points: the SilverCloud platform is configured such that a review period begins with the client completing one set of clinical measures (that is, PHQ-9/GAD-7) that are assigned to them by their clinical supporter. Review periods can therefore be seen as a proxy for time, on average 1.8 weeks in duration (see Fig 1 in S1 Appendix), though this can vary from client to client (SD = 0.24). At the end of the review period, the clinical supporter reviews the clinical measures and all other client interactions with the iCBT program. Based on their assessments, they provide personalised feedback to clients via an online message or telephone contact. At the end of this process, clinical supporters assign questionnaires for a new set of clinical measures to the client, which marks the start of the following review period. We defined the end of treatment as a total of eight review periods into the program, which equates to an average of 13 weeks from the start of treatment (first review). We chose eight review periods as the upper limit since, although some users may engage with the platform beyond this time, they are no longer guaranteed a review by clinical supporters.

Taking the data described above as input features, our objective is the early prediction of treatment outcomes. Within NHS IAPT services in the UK, the “reliable improvement” outcome metric presents a core performance metric for determining treatment success. Reliable improvement reflects a significant positive change in client symptoms, based on the reliable change index by Jacobson and Truax [38]. It is defined as a decrease in total PHQ-9 score of 6 or more points, a decrease in total GAD-7 of 4 or more points, or both at the end of the user’s program (Table 3) [39]. Reliable improvement is chosen as our outcome of interest as the definition indicates *real* improvement in symptoms, exceeding change that can be accounted for by measurement error. In addition, reliable improvement moves the assessment of clinical change from the group mean to the individual being treated.

**Table 3:** Outcomes of interest definitions.

Outcome of Interest	Definition
Reliable improvement in depression	Decrease in PHQ-9 by ≥6 points with no increase ≥4 in GAD-7 at the end of the programme (8 review periods);
Reliable improvement in anxiety	Decrease in GAD-7 by ≥4 points with no increase ≥6 in PHQ-9 at the end of the programme (8 review periods)

**Fig 2:**
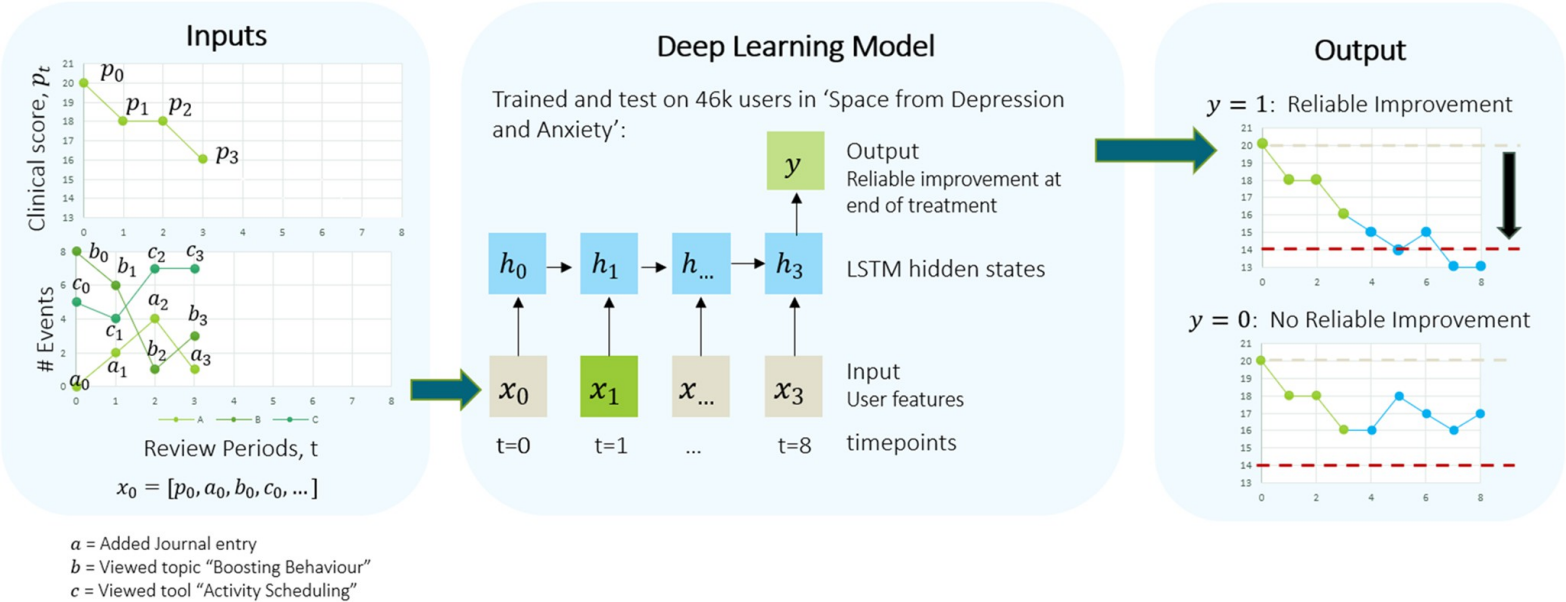
Deep learning architecture to predict reliable improvement in depression or anxiety. We used counts of engagement events with the SilverCloud platform and early clinical scores (PHQ-9 and GAD-7 for depression and anxiety, respectively) as the input features. Using these inputs, we trained a Recurrent Neural Network (RNN) to predict whether a client has reliable improvement at the end of the treatment period. We used LSTM hidden states to represent input features at each time point.

For those users with measurements at fewer than eight review periods. we base the outcome on the last available measurement; we use last-observation-carried-forward (LCOF) imputation to handle label censoring due to user dropout. Evaluation of predictive performance is based only on time points up to and including the last available measurement for each user. Overall, approximately 26% of users included in this study experience reliable improvement in PHQ-9 and 38% experience reliable improvement in GAD-7, where the majority of these are users with higher (moderate to severe) baseline symptom scores (see Fig 2 in S1 Appendix). As evidenced by our findings later, there is necessarily a trade-off between time to prediction and predictive accuracy, whereby earlier predictions may be most helpful to clinical decision-making yet more prone to model uncertainty and prediction errors.

### Machine learning for outcome prediction

We use a deep learning framework to train and test the prediction of reliable improvement in depression and anxiety symptoms for a given client over the course of treatment. Specifically, we consider deep recurrent neural networks (RNNs). RNNs are a class of deep learning methods designed to model sequential data or time series data. Unlike traditional feedforward neural networks, RNNs have connections between the neurons that form directed cycles, allowing them to maintain a hidden state or "memory" of past inputs [40]. This makes them particularly well-suited to learning patterns and dependencies in sequences, and these models have been shown to achieve state-of-the-art performance in many applications with sequential data, including clinical time series [30, 41]. In contrast to classical linear models such as logistic regression, or iterative variants [20, 28], deep RNNs provide a natural framework for extracting patterns from high-dimensional, unstructured data, capturing long-term temporal dependencies in observations, and making predictions with variable length inputs [40]. This makes them well-suited to modelling rich, sequential clinical measurement and iCBT program interaction data. Additionally, the availability of a large-scale digital dataset makes it feasible to train and reliably evaluate these more data-intensive models.

We investigate RNN models trained on our outcomes of interest (reliable improvement in PHQ-9 and GAD-7, respectively), using four different combinations of the feature sets (Table 2):

1. Counts of *each* of the 1133 unique iCBT program interactions (**I**);
2. Early total PHQ-9 and/or GAD-7 questionnaire scores (**Q**);
3. Answers to individual PHQ-9 and GAD-7 questionnaire component question items (**Qc**); and
4. Combination of (i) iCBT program interactions and (ii) total clinical scores (**I+Q**).

Using these inputs, we split our data at random 70:20:10 into training, validation, and test sets, respectively, ensuring stratified samples by outcome label. We then trained a three-layer RNN to predict a binary outcome (whether a client will show reliable improvement at the end of the eight review periods (Fig 2)) using a many-to-one architecture, mapping a variable-length sequence of input data points to produce a single output or prediction. We used a 50-dimensional hidden layer with long-short term memory (LSTM) units to encode client features at each time point. Prediction models were trained independently for depression (PHQ-9) and anxiety (GAD-7) outcomes.

We also implemented several benchmark classification methods to compare against the predictive performance of the RNNs: logistic regression (LogR), random forests (RF), and gradient boosting classifiers (GBMs). These models form the basis of iCBT outcome prediction models in existing literature [24, 29, 42], and have been shown to achieve state-of-the-art performance. In each case, these models take as input feature vectors of length 16, comprising the total PHQ-9 or GAD-7 scores at all time points available so far (0 where not available to avoid imputation bias in the feature space and treating measurements as missing-at-random) up to a maximum of 8 measures, and 8 indicator variables, denoting whether a measure is, in fact, available at that time. This allows us to make a fair comparison with RNNs trained on total scores alone (RNN-Q). Unlike RNNs, however, these models do not *explicitly* use the input data’s sequential nature. As an additional benchmark, we implement exponential moving averages (EMA), a naïve trend-following algorithm, for predicting reliable improvement. The EMA forecasts the expected subsequent clinical measurement and uses this to generate a binary outcome label. The RNNs and benchmark models were built on the training set. Feature and model selection was based on the results from the validation set. The final model was evaluated on the held-out test set and several external cohorts. The models were trained and tested on the AzureML infrastructure in Python 3.7. RNNs were implemented using PyTorch.

## Results

To investigate the most informative features in the prediction of treatment outcomes, we trained RNN models on the four different feature sets (I, Q, Qc, I+Q) described previously and reported the overall performance of these models on our validation dataset (Table 4). The RNN based on input features combining total clinical questionnaire scores (Q) and iCBT program interaction data (I) achieves the highest accuracy for both PHQ-9 (RNN-I+Q = 83.9%) and GAD-7 (RNN-I+Q = 79.84%), while interaction data alone (I) is the poorest predictor of reliable improvement (RNN-I_PHQ-9_ = 71.15%, RNN-I_GAD-7_ = 60.93% accuracy). Interestingly, the best-performing models (RNN-I+Q), built on significantly higher-dimensional data, only marginally outperform the RNNs built on total clinical scores alone (RNN-Q). Similarly, using individual score components (RNN-Qc) instead of the total score does not provide any gains to the prediction task, yielding slightly lower accuracies for PHQ-9 and GAD-7 outcome prediction.

**Table 4:** Optimizing data featurization.

	*PHQ-9* (*n_users_ = 8993*)						*GAD-7* (*n_users_ = 9240*)					
	Acc	Sens	Spec	90%	95%	97%	Acc	Sens	Spec	90%	95%	97%
*RNN-Q*	83.75	49.85	97.55	61.91	53.08	50.66	78.86	61.1	91.08	61.1	54.2	47.62
*RNN-Qc*	83.66	50.25	97.26	62.44	54.28	50.65	78.8	62.68	89.89	62.53	55.17	48.0
*RNN-I*	71.15	1.63	99.46	18.98	10.44	6.51	60.93	20.67	88.65	18.33	10.1	6.53
*RNN-I+Q*	83.90	55.11	95.63	65.25	56.01	51.3	79.85	65.57	89.68	65.12	55.33	47.46

The table shows RNN validation set performance (in terms of accuracy (Acc.), sensitivity (Sens.), specificity (Spec.), along with sensitivity at fixed thresholds of 90%, 95% and 97% specificity respectively) with different feature inputs, averaged over all prediction timepoints. The combination of features considered are: overall PHQ and GAD scores (Q; input dimension = 1), individual score components (Qc; input dimension– 9/7), engagement alone (I; dimension = 1133) and engagement with total clinical scores (I+Q; dimensions = 1134).

To understand the clinical applicability of the predictive model, we also report performance at various operating points trading off sensitivity and specificity, focusing on regions of high specificity (minimizing false positives, where clients are wrongly predicted to improve). While RNN-I+Q consistently performs best for PHQ-9 and GAD-7 improvement prediction, gains over RNN-Q are limited, particularly in regions of high specificity. This, together with the fact that RNN-Q is a much more parsimonious model with fewer data requirements, motivates the use of RNN-Q over RNN-I+Q in practice. We, therefore, restrict ourselves in subsequent analysis to models taking only the total questionnaire scores as input (RNN-Q).

**Fig 3:**
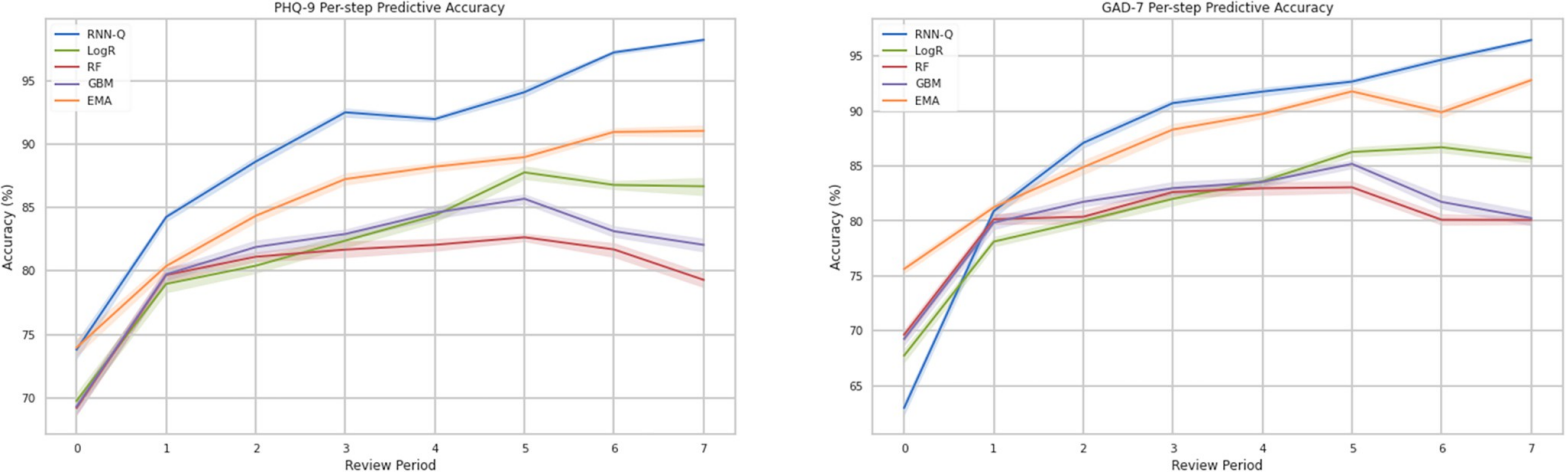
RNN-Q model performance on test dataset by review period plotted against benchmarks for both PHQ-9 and GAD-7. We evaluate our predictions from these two models by stratifying according to the number of measurements available at the time of prediction, to visualize their performance over time. Benchmarks of logistic regression (LogR), random forests (RF), gradient boosting machines (GBM) and exponential moving averages (EMA) are plotted alongside RNN-Q model performance for PHQ-9 (left) and GAD-7 (right), with bootstrapped confidence intervals. All of the above models have access to all prior clinical measures (up to the review period at prediction time). Original files can be found in S1 Dataset.

To further compare the performance of the RNNs with three benchmark machine learning models: LogR, RF, GBMs, as well as the results of the EMA, Fig 3 plots the predictive accuracy of each of these models over time. We find that the RNN-Q consistently outperforms other ML benchmarks, with gains increasing with time into the program. While EMA—which explicitly models temporal trends—performs reasonably well (outperforming logistic regression and other machine learning methods), RNN-Q consistently achieves higher accuracies when three or more clinical measurements are available at the time of prediction.

### Model generalization and subgroup performance

The overall predictive performance of RNN-Q on our test set for PHQ-9 (82.37%) and GAD-7 (77.92%) is summarized in Table 5; further details can be found in S2 File. Again, with the lens of clinical applicability, we investigate the model performance (i) across different client symptom subgroups; and (ii) at time points for which predictions will be made available. We found that prediction accuracy is higher for clients with mild depression and anxiety symptoms (with initial PHQ-9 between 0–8 or initial GAD-7 between 0–6) than those reporting moderate to severe baseline symptoms, which may be due to the class imbalance in this subgroup: few users with low initial scores will go on to meet the change criteria for reliable improvement. We also observed a largely monotonic improvement in the RNN’s performance over time, with accuracy consistently above 87% across all client populations after three review periods. More detailed evaluation of model performance is presented in S3 File.

**Table 5:** RNN-Q test performances for PHQ-9 and GAD-7 at different time intervals and across user subgroups.

		Accuracy		AUROC		Sens. at 95% Spec.	
		All t	t≥3	All t	t≥3	All t	t≥3
PHQ-9 *(n*_*users*_ *= 4493)*	**Overall**	**82.37**	**87.77**	**0.849**	**0.895**	**50.54**	**66.1**
	Mild	93.75	93.94	0.835	0.890	42.98	62.35
	Moderate	79.27	85.93	0.806	0.871	48.16	52.56
	Severe	75.01	84.26	0.814	0.877	47.7	51.62
GAD-7 *(n*_*users*_ *= 4617)*	**Overall**	**77.92**	**87.37**	**0.849**	**0.910**	**52.66**	**70.27**
	Mild	89.19	90.88	0.843	0.895	48.43	72.16
	Moderate	75.41	86.49	0.817	0.906	49.58	69.35
	Severe	73.69	86.23	0.826	0.895	50.5	54.47

RNN-Q test performance for accuracy, AUROC and sensitivity and 95% specificity, overall and by subgroups according to initial severity of symptoms. Performance is reported for both predictions at all time points t, and for only t ≥ 3 (when a minimum of three clinical measures are available for prediction).

**Fig 4:**
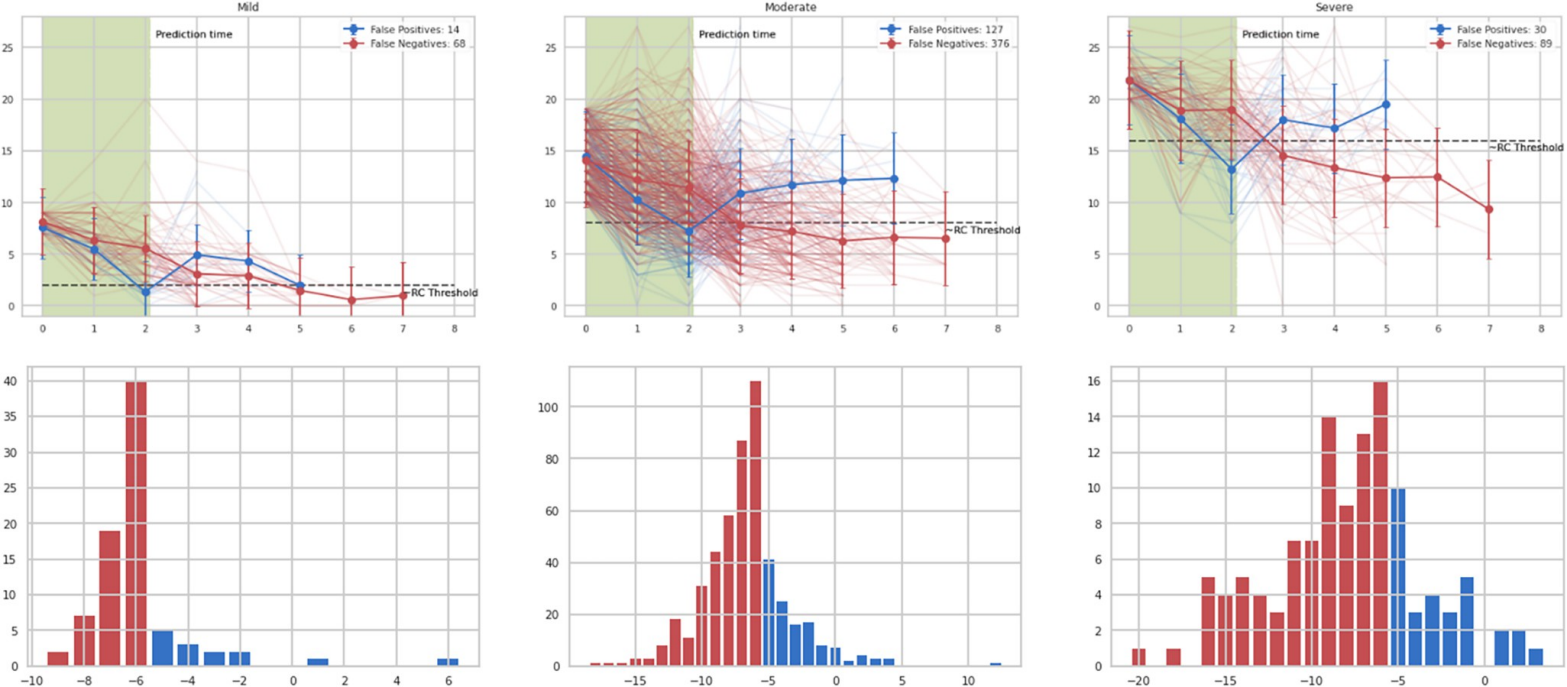
PHQ-9 error analysis. Top three figures (left-to-right) show the symptom trajectories for each of the errors, along with the mean trajectory, in bold. Bottom three figures (left-to-right) show the distribution of final changes in overall symptom score across the errors. Shaded in green are the measurements available during prediction (t = 3). False negatives (red) occur when symptom improvement is marginal in the initial weeks. False positives (blue) occur where a sharp fall in client-reported symptoms is observed before the prediction point, but scores rise again towards the baseline. In both modes of error, the true change in client scores is clustered around -6 (the threshold for reliable improvement), as would be expected, and tails off rapidly away from this threshold.

**Fig 5:**
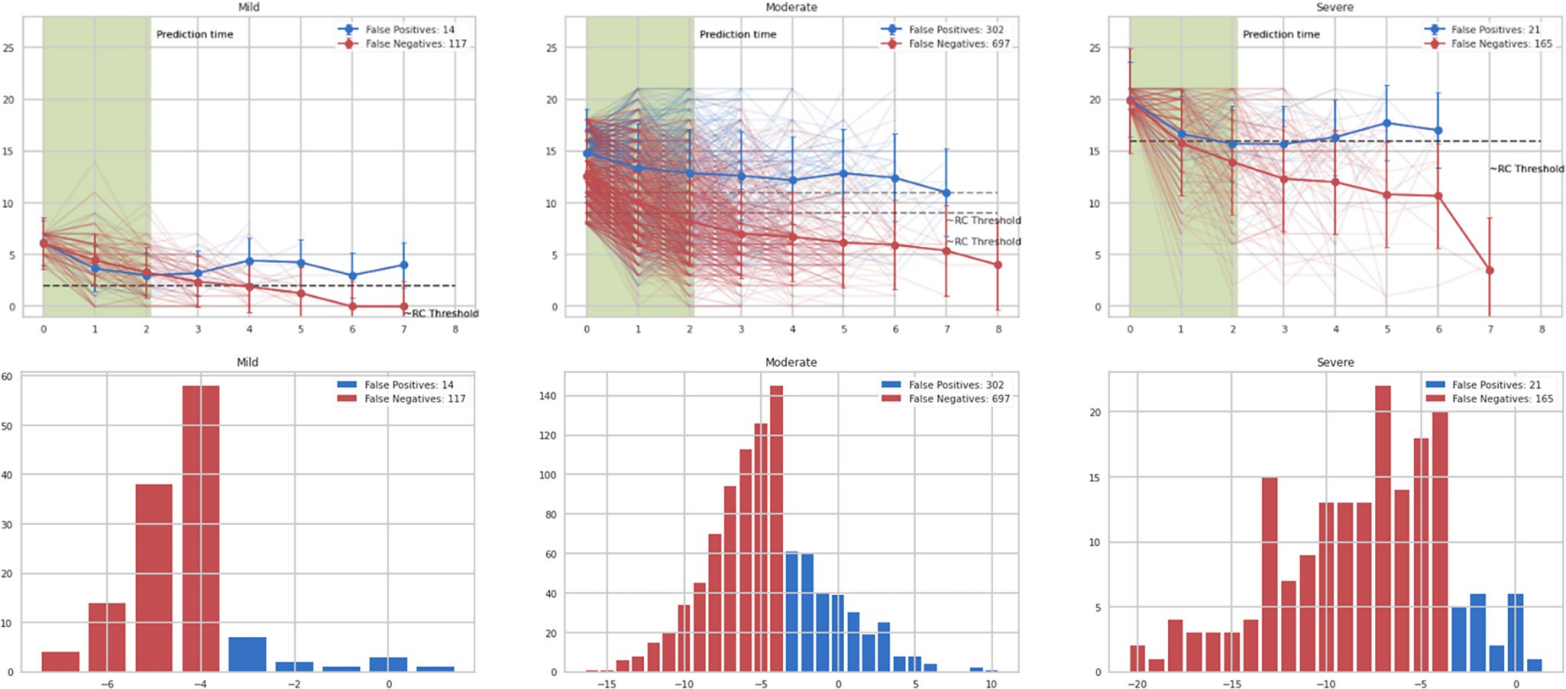
GAD-7 error analysis. Top three figures (left-to-right) show the symptom trajectories for each of the errors, along with the mean trajectory, in bold. Bottom three figures (left-to-right) show the distribution of final changes in overall symptom score across the errors. In both false positives (blue) and false negatives (red), the true change in client scores is clustered around -4 (the threshold for reliable improvement), as would be expected, and tails off rapidly away from this threshold.

The solid predictive performance for both PHQ-9 and GAD-7 outcomes symptom subgroups motivates closer examination of the prediction errors that occur to interpret model behaviour. Figs 4 and 5 (for PHQ-9 and GAD-7, respectively) show the prediction errors for the models on the validation dataset, at review period t = 3 (i.e., given three measures so far in the program), for subgroups with different baseline symptom severity. We find that errors are generally concentrated in clients for whom the actual change in scores by the end of the program is near the threshold for reliable improvement, likely within the margin of measurement error; this is particularly pronounced in the mild/moderate groups. In addition, looking at the typical trajectory for false negatives in reliable improvement prediction for PHQ-9, we find that this typically occurs when there has been little improvement in scores until the point of prediction, followed by a sudden drop from the fourth measurement onwards. Conversely, false positives (which are in the minority of errors) occur in trajectories that see large improvements early in the program, but regress to much more modest improvement over the baseline as time progresses. In both cases, these are very much plausible errors—large drops or reversals in trends are naturally harder to predict—and provide further validation of model behaviour.

Finally, we evaluated the models performance on several external datasets across UK and US settings and for different iCBT programs. Table 6 provides an excerpt of the main findings; full results can be found in S4 File. These results are again compared against model performance on the internal test dataset and demonstrate the robustness of the achieved models across different health services, geographic locations, and iCBT programs. While demographic characteristics were largely unavailable, we also include model performance across age, gender and ethnicity subgroups on a small external cohort for which this data was collected and show that high accuracy is maintained across these subpopulations.

**Table 6:** Overview of model performance across external cohorts.

*External Cohort Performance (all t)*	*PHQ9*				*GAD7*			
	*n*_*users*_	*Acc*.	*Sens*.	*Spec*.	*n*_*users*_	*Acc*.	*Sens*.	*Spec*.
*US single service provider* *Multiple programs (D1)*	2585	86%	48%	97%	2572	78%	64%	87%
*UK multiple providers; post development* *“Space from Depression & Anxiety” (D2)*	31149	83%	50%	96%	31057	77%	65%	87%
*UK single service provider* *Multiple programs (D3)*	10625	82%	51%	96%	10610	77%	64%	87%

Model performance on external cohorts from varying time periods, geographic locations (US, UK), health service providers and iCBT programs.

## Discussion

The availability of coherent, high-fidelity digital engagement data (with click-level interactions logged for users all with access to the same treatment) and clinical outcome measures, as part of one of the largest datasets available for a clinical sample receiving internet-delivered cognitive behaviour therapy (iCBT), supported the development and evaluation of a deep learning framework for the prediction of treatment outcomes. This allowed us to counter many modelling limitations inherent to the classical statistics and machine learning approaches seen in prior work. Our best-performing models, based on total questionnaire scores of PHQ-9 and GAD-7, consistently predicted reliable improvement changes in clinical symptoms with above 87% accuracy and 0.89 AUC early during treatment (after three or more review periods). Notably, we achieve above 66% sensitivity for PHQ-9 and 70% for GAD-7 when designing for over 95% specificity (a false positive rate of 1 in 20), which is crucial to the utility of these predictions in clinical decision-making. While the limited prior work with large-scale observational iCBT data and variations in client selection criteria or outcome of interest makes direct comparisons challenging (most recently, Bone et al. achieved an AUC of 0.81 at a comparable stage in treatment) [24], to our knowledge this is the best performance achieved in the dynamic prediction of psychological treatment outcomes to date–especially for predicting clients who are at risk of not achieving RI [17]–and the first use of deep learning for outcome prediction from a dataset treating clients using iCBT alone (for other iCBT-based explorations in this space see Boman et al. [43]). Our RNN-based approach outperformed several competitive benchmarks and maintained predictive accuracy across multiple large-scale external cohorts, supporting its utility in outcome prediction across diverse populations, different iCBT programs, and geographical locations (UK, US). This latter work increases the ecological validity of the DL model, work that has not been achieved by previous developments in outcome prediction in this area [17].

Having explored different feature sets in training RNN models, we found that the RNN based on input features combining total clinical questionnaire scores (Q) and iCBT program interaction data (I) achieved highest accuracy, outperforming those built on either one dimension or using individual score components instead of total scores. This may be attributed in part to increased overfitting, as well as the significant heterogeneity across the client population in symptom profiles and, in turn, which symptom changes contribute most to reliable improvement for different client subgroups, making it challenging to outperform the use of aggregate scores in predicting the defined outcomes. We also found in our analysis that iCBT program interactions, though a rich source of data on the platform, yield noisy predictors when taken alone as input, and contribute little additional predictive value to use of just clinical measures. One reason may be that program interactions, as a proxy for treatment engagement, contribute to outcomes via psychological change. This contribution itself will be represented within the outcome data of previous weeks, limiting the value of including interaction data separately. There may also be opportunities for different approaches to learning representations for program interactions. In our work, we encoded these as 1133 unique section-action-content sequences, which may not be the right granularity to extract meaningful patterns. Possible directions for future work include defining interactions based solely on the treatment section, grouping therapy contents by overall theme, or identifying the unique treatment components that contribute to behaviour change.

On the other hand, information may be lost by representing program interaction solely on aggregate counts: modelling instead of temporal patterns in platform use within each review period may be more informative; the RNN architecture provides a flexible, high-capacity framework for such exploration. Furthermore, we must acknowledge that within this digital treatment set-up, it can be harder to separate between a client’s interactions with the digital platform and their engagement with treatment (i.e., client ‘views’ of therapy-based *Content* pages themselves carry little information about the extent to which the client may attentively read and understand that content). While the relationship between platform interactions and outcomes is likely complex [44], active engagement with the iCBT program is essential in contributing to change. It is well understood and often targeted by clinicians to help improve client outcomes [45]. Thus, further research into including treatment interaction for explainable predictions is warranted.

Results illustrate how the accuracy of the dynamic outcome predictions with our RNN models increases with time. While early prediction of client outcomes may be most informative to clinical practice, later predictions are more reliable. Given this trade-off, we suggest that predictions could be made available to PWPs after a minimum of three review periods (typically within the first six weeks), from which point our models consistently achieve above 87% accuracy and 0.89 AUROC whilst leaving a clinically actionable time window for therapy that typically lasts eight review periods. However, at the same time, this restriction to a minimum of three review periods means that a significant proportion of clients (almost 60% based on training data distribution), who might drop out, change or complete the program ahead of this time, would not be predicted for in practice. Future studies are needed to explore appropriate “prediction accuracy–client inclusion” trade-offs, and how they impact clinical utility during the provision of support, especially in the broader context of feedback-informed psychotherapy, where expected treatment response models typically require just two assessments [14].

In any clinical application of machine learning, we need to be mindful of the potential harm of prediction errors. In the context of feedback provision within iCBT, there is general agreement among clinical experts that false positives must be minimized, as these constitute clients that need extra help and may be at risk of not receiving it, potentially delaying recovery. On the other hand, false negative predictions mean that the model predicts that a client not to achieve reliable improvement when in fact, they do. False negatives can disrupt a client’s treatment journey if they cause unnecessary or unhelpful adaptations–for example, the client is referred to more intensive care when they didn’t need to be–leading to poorer client experiences and misdirection of limited resources. However, for most negative RI predictions (whether true or false), clinicians described that they would work harder to identify the clients’ difficulties and treatment needs, meaning that false negative errors may not come to weigh in as much as false positives (not receiving extra help when needed), which is why we chose to restrict our model specificity to above 95% (low false positive rate), at the expense of a higher false negative rate. Despite such adjustments and overall low error rates, it remains paramount that clinicians are carefully educated about the probabilistic nature of prediction models and their potential for errors so that they can appropriately interpret and use the provided information. This is particularly important given the heterogeneity in treatment response and potential fluctuations at an individual level, particularly in cases where abrupt changes in symptom trajectories may occur, requiring clinicians to balance assessments of the predicted outcomes with their professional expertise. Future research must carefully assess the appropriateness and real-world implications of this chosen threshold/ trade-off and guide necessary adaptations.

### Limitations

There are multiple ways in which outcomes can be defined to inform clinical decision-making including risks of symptom deterioration; chance of recovery or remission; specific score change; mental health trend; or, as in this case, a significant reduction in symptoms. The focus on reliable improvement was a deliberate choice as it is an established metric for measuring the success of treatments delivered in the IAPT program, the context in which this work is focused. However, we acknowledge that there are controversies and a lack of agreement in determining a good threshold for assessing improvement in mental health. Further, we decided to predict a score change ‘threshold’ rather than the change in the score itself (a more sensitive measure). We found the threshold to provide a less ambiguous signal that a client might be at risk of not improving significantly (negative RI prediction). Furthermore, in a subsequent, planned deployment study (see below), we decided not to present predictions for patients with scores below ‘caseness’, for whom it is more difficult (numerically) to achieve RI, which makes negative RI predictions most probable. Yet, those predictions are less likely to indicate poor patient progress or need for more support, as these individuals are already in the desired score bounds of recovery or remission.

Furthermore, with the prediction models based solely on clinical scores, we acknowledge that no further insights are given into the potential mechanisms of suggested treatment failures, nor are concrete actions proposed for how clinical supporters may address any treatment difficulties. Nonetheless, and in line with other recent research on the dynamic prediction of treatment outcomes [24], we consider a DL tool with high accuracy for predicting outcomes that serve as a ‘prompt’ for clinical review to significantly enhance response rates to treatment by enabling accurate and timely feedback to clinical supporters to improve clinical decision making (i.e., considering stepping clients-up or -out to alternative treatments, and tailoring treatment to client preferences and expectations) [17]. As such, this work responds to calls by intervention researchers to systematically evaluate client response to treatment to determine if the course is progressing as expected and, if not, to modify or change treatments as suitable [46].

Another limitation inherent to the observational setting of this study is that like previous works [17], our analyses rely on the use of last observation carried forward methods, where the last observed measure for each user is taken to determine the post-treatment outcome, which introduces label error for those clients who drop out before the end of the treatment, potentially conflating predictive performance. In S3 File, we quantify this gap by evaluating performance on only those users for which eight or more clinical measurements are available. However, given that our objective here is to evaluate the utility of dynamic predictions in clinical decision-making for *all* users with highly heterogenous platform use (many of whom may complete treatment content or achieve reliable improvement before the full intended length of treatment), our priority was to be as inclusive as possible in our analysis, requiring a minimum of just two clinical measurements. Additionally, there is considerable scope for further work to better understand outcome prediction over the course of treatment with this richer dataset. For example, previous psychotherapy research has shown how clients who improve early in therapy and make sudden mental health gains [47] tend to show the best outcomes. These effects are often demonstrated in conventional psychotherapy and have also been found for briefer, low-intensity CBT treatments [17]. If early change is a significant predictor of outcomes, future work could assess if there are any differences in model performance when predicting early versus later improvers, suggesting higher accuracy rates for those early improvers.

### Towards real-world implementation

In addition to considerations of model robustness, several practical implications need to be realized to introduce machine learning insights successfully into real-world clinical workflows [48]. We regard iCBT as particularly well-suited to the adoption of these methods. In these digital health services, outcome data is already routinely collected and monitored, and clinical supporters are responsible for examining this outcome data in the context of iCBT treatment by using data dashboards to review client progress and support case management. It is thus more straightforward to present relevant insights to clinicians, focusing on the added value of these techniques and appropriate practices for their use, rather than implementation barriers. In a separate paper [49], we report initial learnings from user research with iCBT supporters that clarified concrete use scenarios and potential concerns about integrating achieved prediction outcome models within iCBT practice. It also highlights how design choices in the supporter user interface and workflow integration can help mitigate the risks of over-reliance on AI outputs.

The integration of the prediction models within the supporter user interface further paves the way for real-world studies to assess the impact of introducing robust and reliable DL methods into clinical practice, intending to support clinicians in making early and necessary treatment adjustments to promote a favourable therapeutic response in the greatest number of clients. Following on from this research, a randomized controlled trial (https://www.isrctn.com/ISRCTN18059067) is being designed to investigate the effectiveness of feedback-informed iCBT treatment through DL algorithms in improving symptoms of depression and anxiety, delving into clinicians’ perception on acceptance of these models, their subjective experience with the model outputs, and ultimately their potential to impact the provision of support and improve clinical outcomes.

## Conclusions

Multiple studies assessing the benefits of feedback-informed therapy (FIT) have demonstrated how providing therapists or clinical supporters who assist the person undergoing treatment with access to feedback on expected client outcomes from treatment can improve treatment success and prevent deterioration, especially in clients with poor prognoses. Expected treatment response models are the methodological standard for assessing treatment outcomes in psychotherapy interventions, with few studies investigating the use of machine learning for the dynamic prediction of desired treatment outcomes. Within iCBT contexts, predictive models have been built on modest and selective samples (ranging from <100 to a few thousand users) and have typically involved post hoc analysis of RCT data and miscellaneous data sources, with mixed results. Reported accuracies range between 55–83%; however, different means of computing those and variations in the underlying treatment programs make any direct comparisons challenging.

This study demonstrates the feasibility of using a deep learning (DL) framework to achieve robust, dynamic outcome prediction models. DL methods achieve state-of-the-art performance in many settings, given their ability to handle high-dimensional inputs and model complex, non-linear patterns in unstructured data with limited feature engineering. Deep RNNs provide a natural framework for modelling and making predictions given sequential data. The models presented here, based solely on routinely collected clinical outcome measures of depression and anxiety symptoms, yield high accuracies early into treatment (>87% after just three review periods), outperforming several other machine learning and advanced statistical methods. They also show generalizability to data from different iCBT programs, geographies, and demographic groups.

Methodological implications for the current work depart from previous limitations in evidence in two ways: first, by using a large-scale dataset in training and validation that closely matches the intended implementation context and second, by employing a high-capacity DL framework to deliver robust, dynamic outcome prediction with high accuracy. The clinical implications of these models in digitally delivered psychotherapy services include enabling the near-term deployment and clinical study of such models within iCBT care that already routinely collect clinical outcome measures and employ digital review practices. This moves the field of precision psychiatry one step closer alongside many paths that seek to enable clinical supporters to prioritize better and adapt real-time interventions for clients with the greatest unmet need.

## Supporting information

S1 File De-identification and pre-processing pipeline. (DOCX)

S2 File Model featurization in benchmarks vs RNN. (DOCX)

S3 File Internal validation and test performance results. (DOCX)

S4 File External validation results. (DOCX)

S1 Appendix **Fig 1**. User distribution and reliable improvement over time. Top figure: Distribution of time from enrolment to nth review; bottom figure: Counts of users at each review period. **Fig 2**. Kaplan Meier curves modelling reliable improvement events in PHQ-9 and GAD-7 for clients with different baseline severity. Survival probability corresponds to the probability that sustained reliable improvement is not achieved by a given timestep in the treatment program. A steeper drop in survival probability can be interpreted as higher rate of reliable improvement in that subpopulation.
(DOCX)

S1 Dataset Benchmark numerical results on validation dataset for PHQ and GAD. These data sets correspond to the results shown in Fig 3 (per-step predictive accuracy).
(ZIP)
