# Supplementary material for: Deep learning for the prediction of clinical outcomes in internet-delivered CBT for depression and anxiety
Source: PLoS One. 2023 Nov 27;18(11):e0272685. doi: 10.1371/journal.pone.0272685 (PMC10681250; doi:10.1371/journal.pone.0272685)
Supplement: S1 File — (DOCX) [file pone.0272685.s001.docx]

# **S1 File. De-identification and pre-processing pipeline.**

As an initial step, we clean the data of personally identifiable data and other data that is not of interest. This involves three processes, all completed in Django using the operational data from the SilverCloud platform:

- Some tables are of no interest to the team building the model or contain data that is private to the platform. These tables are dropped.
- Some tables contain a mixture of relevant and irrelevant/private data. These tables have the private data zeroed or otherwise nulled.
- For personal data there are three approaches taken
  - Sometimes the data can be loosened so that is no longer identifiable. For example, the date a client joined becomes the year they joined.
  - Timestamps are turned from actual datetimes into relative times, i.e. the number of seconds since the client joined.
  - The unique id for each client, event, etc. if given an isomorphic translation so that it differs from the original but can still be traced back on the platform if data anomalies need to be explained.

This data is then filtered to remove:

- - Supporters from the client data
  - Any test clients used to feature test the platform
  - Clients in services that are now discontinued
  - Clients with malformed identifiers (there are a handful of clients with nonsensical module identifiers etc
  - Clients with negative dates (for a handful of clients the process mapping datetimes to seconds since start must have failed)

Finally, the data is prepared for use within machine learning models by:

- - Filtering to the programme of interest (Depression and Anxiety in our case)
  - Filtering to certain time periods (e.g. up to review period 8)
  - Restricting to numerical columns
  - Producing counts of event data over different time periods
  - Producing features, such as reliable improvement, for the models to learn against.
