# Supplementary material for: Deep learning for the prediction of clinical outcomes in internet-delivered CBT for depression and anxiety
Source: PLoS One. 2023 Nov 27;18(11):e0272685. doi: 10.1371/journal.pone.0272685 (PMC10681250; doi:10.1371/journal.pone.0272685)
Supplement: S2 File — (DOCX) [file pone.0272685.s002.docx]

# **S2 File. Model featurization in benchmarks vs RNN.**

The machine learning benchmarks used in this study for comparison against RNNs taking total scores as input (logistic regression, random forests and gradient boosting machines) take 16-dimensional input features (8 score features and 8 indicator variables) visualized by the following. Each user is then represented by *n* samples, where *n* is the number of reviews available for that users, each with the same target label (based on RI by the final time step):


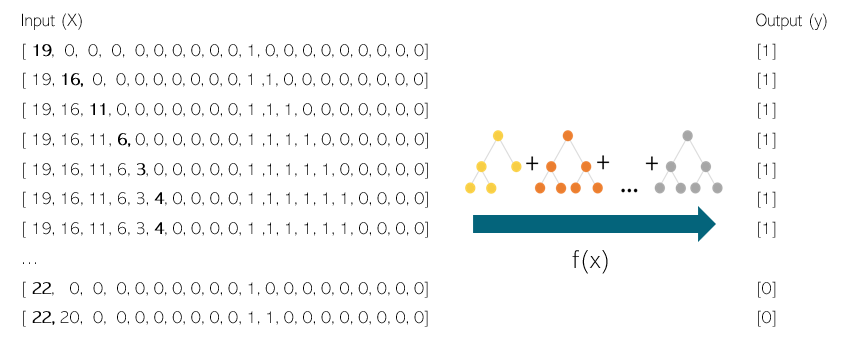


**Fig A. Score trajectory featurization for baseline models (LogR, RF, GBM).**

The RNN handles this variable-length sequence paradigm and many-to-one mapping in a more natural way: during training, we use the label at the final time step and propagate backwards, and at test time, we use as many measures as we have available and output a prediction at that timestep.


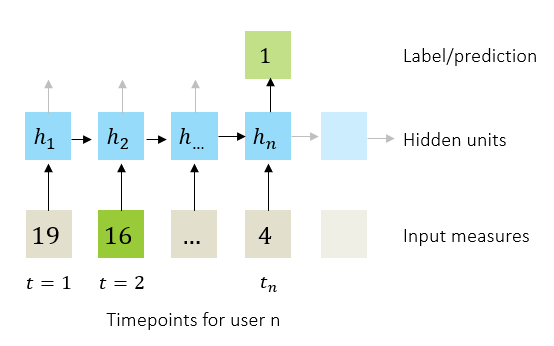


**Fig B. RNN many-to-one mapping.**

The presented RNN architecture comprised an input layer with dimensionality ranging from 1 or 1134 (depending on the choice of features), a hidden layer of dimension 50 with LSTM units, and a 2-dimensional output layer giving class probabilities (for 0 and 1 respectively), followed to a linear softmax to obtain the final binary prediction for reliable improvement. The RNN was trained using ADAM with a dropout probability of 0.4 and cross-entropy loss. For all models, the maximum number of timesteps per user is 8.

Lastly, the EMA (exponential moving averages) benchmark is a type of moving average that places greater weight on the most recent points. Here, we set the half-life to be 0.5: we forecast a score for the next review period by taking a weighted average of all measurements so far, where the last known score is weighted twice as highly as the one before, and so on. If the difference between this predicted score and baseline score is greater than the threshold for reliable improvement, we obtain a positive prediction for reliable improvement.

**Hyperparameter optimization:** For the RNNs, we ran a hyperparameter sweep over the number of LSTM hidden units and dropout probability, with model selection based on highest accuracy on the validation set for models across different input features. For the exponential moving averages baseline, we swept over different values of the half-life term, using the same criteria to choose the best model. We also ran simple hyperparameter sweeps over L1/L2 regularization penalties for logistic regression, and maximum depth + minimum leaf node size for the tree-based models (RF/GBM), but found that these had minimal impact on model performance.
