# Supplementary material for: Deep learning for the prediction of clinical outcomes in internet-delivered CBT for depression and anxiety
Source: PLoS One. 2023 Nov 27;18(11):e0272685. doi: 10.1371/journal.pone.0272685 (PMC10681250; doi:10.1371/journal.pone.0272685)
Supplement: S3 File — (DOCX) [file pone.0272685.s003.docx]

# **S4 File. Internal validation and test performance results.**

**Table A.** **Internal data partitions.**

| Partition | N | N_phq | N_gad | Description |
| --- | --- | --- | --- | --- |
| **All** | 45876 | 45352  Minimal (N, %): 2742 (6.1%)  Mild: 10145 (22.6%)  Moderate: 14050 (31.3%)  Moderately-severe: 11031 (24.5%)  Severe: 6976 (15.5%) | 45756  Minimal (N, %): 3130 (6.8%)  Mild: 12420 (26.9%)  Moderate: 14786 (32%)  Severe: 15826 (34.3%) | All data available during model development: UK users enrolled in Programme 41 (Space from Depression & Anxiety), January 2015 - March 2019 |
| **Train** (70%) | 31982 | 31866 | 31899 | Used to train RNN and benchmark models |
| **Validation** (20%) | 9263 | 8993 | 9240 | Used for model comparison, model selection and error analysis (Tables 3,4; Figures 3,4) |
| **Test** (10%) | 4631 | 4493 | 4617 | Holdout in-distribution test set for evaluation of chosen model (Table 5) |


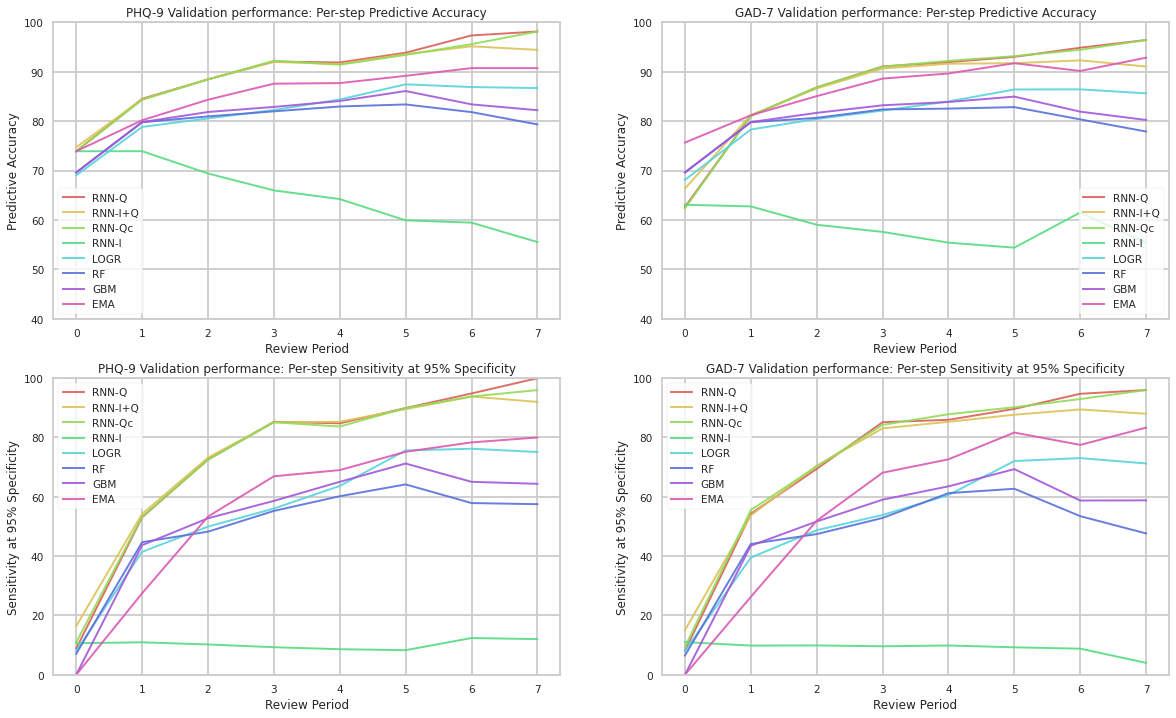


**Fig A.** **Validation set performance over time for different RNN featurizations (alongside benchmark models).** Validation set performance is shown in terms of predictive accuracy (top two figures) and sensitivity at fixed specificity of 95% (bottom two figures) for PHQ-9 and GAD-7 respectively, where the latter metric is a more balanced indicator of the expected performance at the operating point we care about in this setting. We see that RNN-Q consistently performs better than the benchmarks investigated, and comparably with RNNs with more complex feature inputs (RNN-Qc, RNN-I+Q). These metrics motivate our selection of RNN-Q for further analysis.

**Table B.** **RNN-Q validation set performance over time: PHQ-9.**

| **PHQ-9** | **N** | **N(RI)** | **Accuracy** | **AUROC** | **PPV** | **Balanced Accuracy** | **Sensitivity** | **Specificity** | **Sensitivity at 95%** |
| --- | --- | --- | --- | --- | --- | --- | --- | --- | --- |
| All | 30090 | 8707 | 83.85 | 0.87 | 89.2 | 73.7 | 49.8 | 97.6 | 53.1 |
| **T3+** | **12104** | **4017** | **90.47** | **0.94** | **91.2** | **87.6** | **78.9** | **96.2** | **80.1** |
| 1 | 8993 | 2345 | 73.92 | 0.71 | - | 50.0 | 0.0 | 100.0 | 8.9 |
| 2 | 8993 | 2345 | 84.53 | 0.85 | 84.4 | 73.3 | 49.9 | 96.7 | 53.1 |
| 3 | 6109 | 1862 | 88.48 | 0.92 | 88.6 | 83.7 | 71.4 | 95.6 | 72.6 |
| 4 | 3491 | 1197 | 92.07 | 0.95 | 92.0 | 90.2 | 84.1 | 96.2 | 85.3 |
| 5 | 1530 | 558 | 91.90 | 0.96 | 93.1 | 90.2 | 84.1 | 96.4 | 84.8 |
| 6 | 684 | 278 | 93.86 | 0.97 | 95.0 | 93.2 | 89.6 | 96.8 | 89.9 |
| 7 | 227 | 97 | 97.36 | 0.98 | 100.0 | 96.9 | 93.8 | 100.0 | 94.9 |
| 8 | 54 | 25 | 98.15 | 1.0 | 100.0 | 98.0 | 96.0 | 100.0 | 100.0 |

**Table C. RNN-Q validation set performance over time: GAD-7.**

| **GAD-7** | **N_total_** | **N_RI_** | **Accuracy** | **AUROC** | **PPV** | **Balanced Accuracy** | **Sensitivity** | **Specificity** | **Sensitivity at 95%** |
| --- | --- | --- | --- | --- | --- | --- | --- | --- | --- |
| All | 30886 | 12593 | 78.85 | 0.87 | 89.2 | 73.7 | 49.8 | 97.6 | 54.2 |
| **T3+** | **12406** | **5569** | **89.26** | **0.94** | **91.1** | **88.8** | **84.3** | **93.3** | **78.1** |
| 1 | 9240 | 3512 | 62.66 | 0.67 | 51.9 | 55.2 | 23.8 | 86.5 | 8.1 |
| 2 | 9240 | 3512 | 81.08 | 0.85 | 89.2 | 77.3 | 61.5 | 93.1 | 54.3 |
| 3 | 6260 | 2694 | 86.84 | 0.91 | 89.2 | 85.9 | 79.0 | 92.8 | 69.4 |
| 4 | 3578 | 1638 | 91.08 | 0.95 | 92.4 | 90.8 | 87.7 | 93.4 | 85.2 |
| 5 | 1566 | 741 | 91.95 | 0.96 | 92.4 | 91.9 | 90.4 | 93.6 | 86.0 |
| 6 | 702 | 357 | 93.02 | 0.96 | 93.8 | 93.0 | 92.4 | 93.6 | 89.6 |
| 7 | 234 | 114 | 94.87 | 0.97 | 94.7 | 94.9 | 94.7 | 95.0 | 94.7 |
| 8 | 56 | 24 | 96.43 | 0.99 | 96.0 | 96.4 | 96.0 | 96.8 | 96.0 |

**Table D.** **RNN-Q test set performance over time: PHQ-9.**

| **PHQ-9** | **N_total_** | **N_RI_** | **Accuracy** | **AUROC** | **PPV** | **Balanced Accuracy** | **Sensitivity** | **Specificity** | **Sensitivity at 95%** |
| --- | --- | --- | --- | --- | --- | --- | --- | --- | --- |
| All | 14987 | 4360 | 82.37 | 0.85 | 84.4 | 72.3 | 48.3 | 96.4 | 50.5 |
| **T3+** | **6001** | **1952** | **87.77** | **0.89** | **84.8** | **84.7** | **76.0** | **93.4** | **66.1** |
| 1 | 4493 | 1204 | 73.2 | 0.71 | - | 50.0 | 0.0 | 100.0 | 6.5 |
| 2 | 4493 | 1204 | 84.33 | 0.85 | 83.6 | 73.9 | 51.7 | 96.3 | 54.7 |
| 3 | 3054 | 935 | 0.88.1 | 0.91 | 86.5 | 83.7 | 72.4 | 95.0 | 72.4 |
| 4 | 1744 | 579 | 89.62 | 0.92 | 86.7 | 87.5 | 81.2 | 93.8 | 74.3 |
| 5 | 749 | 263 | 85.45 | 0.89 | 80.1 | 83.7 | 78.0 | 89.5 | 63.5 |
| 6 | 330 | 125 | 84.24 | 0.90 | 79.7 | 83.1 | 78.4 | 87.8 | 68.8 |
| 7 | 97 | 41 | 77.32 | 0.89 | 77.1 | 75.8 | 65.9 | 85.7 | 56.1 |
| 8 | 23 | 9 | 82.61 | 0.88 | 77.8 | 81.7 | 77.8 | 85.7 | 0.0 |

**Table E.** **RNN-Q test set performance over time: GAD-7.**

| **GAD-7** | **N** | **N(RI)** | **Accuracy** | **AUROC** | **PPV** | **Balanced Accuracy** | **Sensitivity** | **Specificity** | **Sensitivity at 95%** |
| --- | --- | --- | --- | --- | --- | --- | --- | --- | --- |
| All | 15386 | 6128 | 77.92 | 0.87 | 76.4 | 75.8 | 65.2 | 86.4 | 52.7 |
| **T3+** | **6152** | **2688** | **87.37** | **0.91** | **88.4** | **86.9** | **70.3** | **91.3** | **70.3** |
| 1 | 4617 | 1720 | 62.59 | 0.66 | 49.8 | 58.9 | 44.7 | 73.3 | 7.0 |
| 2 | 4617 | 1720 | 80.66 | 0.84 | 85.1 | 76.1 | 58.3 | 94.0 | 52.9 |
| 3 | 3137 | 1343 | 86.36 | 0.91 | 88.6 | 85.3 | 78.2 | 92.5 | 69.2 |
| 4 | 1785 | 796 | 89.08 | 0.92 | 89.0 | 88.8 | 86.2 | 91.4 | 76.4 |
| 5 | 766 | 366 | 87.08 | 0.91 | 86.6 | 87.0 | 86.3 | 87.8 | 62.6 |
| 6 | 338 | 167 | 88.17 | 0.92 | 87.1 | 88.1 | 89.2 | 87.1 | 72.5 |
| 7 | 99 | 53 | 87.88 | 0.91 | 90.1 | 88.0 | 86.8 | 89.1 | 0.0 |
| 8 | 23 | 13 | 86.96 | 0.88 | 85.7 | 86.2 | 92.3 | 80.0 | 0.0 |


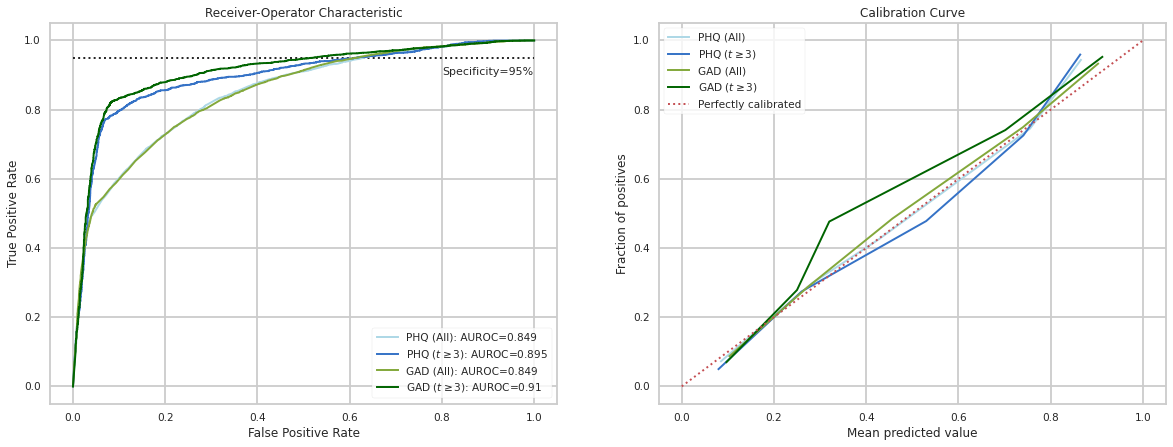


**Fig B.** **Overall RNN-Q test performance for prediction of depression (PHQ-9) and anxiety (GAD-7) outcomes.** The graphic shows: ROC curve (left), Calibration curve (right). For the ROC curve, prediction models that give curves closer to the top-left corner indicate a better performance. In an optimal case, this would result in an AUROC (Area under the ROC curve) score of 1. For the calibration curve, we can see that the predicted probabilities for reliable improvement output by both the PHQ-9 and GAD-7 models correspond closely to the true fraction of positive outcomes in the data (that is, near perfect calibration, represented by the line *y=x*).


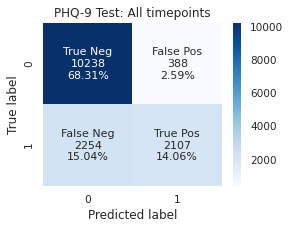

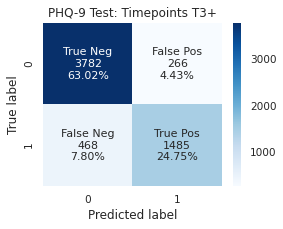


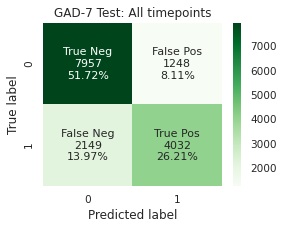

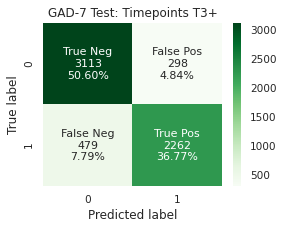


**Fig C. Confusion matrices evaluated on test set for the predictions.** For PHQ-9 predictions, the top-left matrix shows all timepoints and the top-right matrix shows timepoints from review period 3 onwards. For GAD-7 predictions, the bottom-left matrix shows all timepoints, and the bottom-right matrix timepoints from review period 3 onwards.


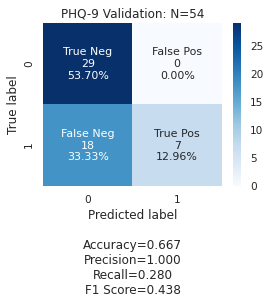

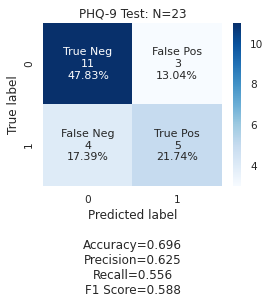


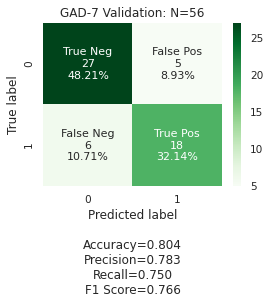

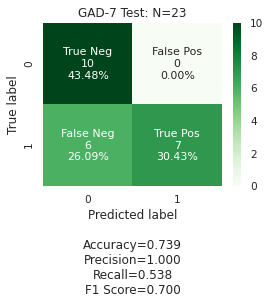


**Fig D.** **Confusion matrices results for only those users with clinical measures available at 8 or more review periods.** Evaluating prediction *at review period 3* of treatment outcome for only those users with clinical measures (PHQ-9/GAD-7) available at 8 or more review periods.
