# Supplementary material for: Deep learning for the prediction of clinical outcomes in internet-delivered CBT for depression and anxiety
Source: PLoS One. 2023 Nov 27;18(11):e0272685. doi: 10.1371/journal.pone.0272685 (PMC10681250; doi:10.1371/journal.pone.0272685)
Supplement: S4 File — (DOCX) [file pone.0272685.s004.docx]

# **S5 File. External validation results.**

Below are performance results of our final model (RNN-Q) on various external validation sets:

1. US single service provider, multiple programs (n=2585/2572)
2. UK multiple providers, ‘Space from Depression and Anxiety’ post model development (n=31149/31057)
3. UK single service provider, multiple programs (n=10625/10610)
4. SilverCloud RCT, program ‘Space from Depression and Anxiety’ (n=82)

## **Data Protection & Privacy**

All SilverCloud users in these datasets have agreed that their de-identified data can be used in routine evaluations for service monitoring and improvement. This permits the analysis of anonymous data for research purposes, and to improve the effectiveness and service tools of the treatment platform. Users were informed that this analysis may include profiling, machine learning or other techniques. Where individual-level data is used, additional safeguards such as anonymisation, use of pseudonyms (pseudonymisation) and limiting the set of individual data used (data minimization) are in place.^[[1]](#footnote-2)^ Users who did not wish to have their data included in analysis could opt-out, and their data was removed.

## **Dataset 1**

**Region:** US

**Total Users**: 2585

**Services:** Single health care service

**Programs:** Multiple

**Treatment Mode:** Supported

**Age:** 18+

**Table A. User programs for dataset 1.**

| **Name** | **n** |
| --- | --- |
| 'Space from Depression & Anxiety' | 1431 |
| 'Space from Anxiety' | 508 |
| 'Space from Depression' | 301 |
| 'Space from Stress' | 345 |

**Table B. Average for all timepoints for dataset 1.**

| **Measure** | **Total Predictions** | **Accuracy** | **Sensitivity** | **Specificity** |
| --- | --- | --- | --- | --- |
| PHQ9 | 7621 | 86% | 48% | 97% |
| GAD7 | 7582 | 78% | 64% | 87% |

**Table C. Timepoints 3 to 10 (when predictions are visible on SilverCloud) for dataset 1.**

| **Measure** | **Total Predictions** | **Accuracy** | **Sensitivity** | **Specificity** |
| --- | --- | --- | --- | --- |
| PHQ9 | 2451 | 88% | 77% | 93% |
| GAD7 | 2438 | 87% | 87% | 90% |

**Table D. PHQ-9 results by timepoint for dataset 1 (n=****2585, total RI=481).**

| **Timepoint** | **Total Predictions** | **RI** | **Correctly Predicted RI** | **No RI** | **Correctly Predicted No-RI** | **Accuracy** | **Sensitivity** | **Specificity** |
| --- | --- | --- | --- | --- | --- | --- | --- | --- |
| 1 | 2585 | 481 | 0 | 2104 | 2104 | 81.00% | 0.00% | 100.00% |
| 2 | 2585 | 481 | 246 | 2104 | 2070 | 90.00% | 51.00% | 98.00% |
| 3 | 1224 | 324 | 222 | 900 | 848 | 87.00% | 69.00% | 94.00% |
| 4 | 677 | 216 | 177 | 461 | 426 | 89.00% | 82.00% | 92.00% |
| 5 | 337 | 120 | 99 | 217 | 200 | 89.00% | 82.00% | 92.00% |
| 6 | 124 | 51 | 44 | 73 | 64 | 87.00% | 86.00% | 88.00% |
| 7 | 49 | 24 | 22 | 25 | 23 | 92.00% | 92.00% | 92.00% |
| 8 | 26 | 10 | 8 | 16 | 13 | 81.00% | 80.00% | 81.00% |
| 9 | 13 | 6 | 6 | 7 | 6 | 92.00% | 100.00% | 86.00% |
| 10 | 1 | 0 | 0 | 1 | 1 | 100.00% |  | 100.00% |

**Table E. GAD-7 results by timepoint for dataset 1 (n=2572, total RI=809).**

| **Timepoint** | **Total Predictions** | **RI** | **Correctly Predicted RI** | **No RI** | **Correctly Predicted No-RI** | **Accuracy** | **Sensitivity** | **Specificity** |
| --- | --- | --- | --- | --- | --- | --- | --- | --- |
| 1 | 2572 | 809 | 315 | 1763 | 1307 | 63.00% | 39.00% | 74.00% |
| 2 | 2572 | 809 | 485 | 1763 | 1705 | 85.00% | 60.00% | 97.00% |
| 3 | 1219 | 518 | 406 | 701 | 639 | 86.00% | 78.00% | 91.00% |
| 4 | 674 | 325 | 291 | 349 | 321 | 91.00% | 90.00% | 92.00% |
| 5 | 334 | 178 | 157 | 156 | 140 | 89.00% | 88.00% | 90.00% |
| 6 | 122 | 72 | 64 | 50 | 42 | 87.00% | 89.00% | 84.00% |
| 7 | 49 | 36 | 33 | 13 | 10 | 88.00% | 92.00% | 77.00% |
| 8 | 26 | 18 | 15 | 8 | 5 | 77.00% | 83.00% | 62.00% |
| 9 | 13 | 10 | 7 | 3 | 1 | 62.00% | 70.00% | 33.00% |
| 10 | 1 | 1 | 0 | 0 | 0 | 0.00% | 0.00% |  |

## **Dataset 2**

**Region:** UK

**Total Users**: 31151

**Service:** Multiple health care services

**Program:** Space from Depression & Anxiety, post training of the model.

**Treatment Mode:** Supported

**Age:** 18+

**Table F. Average for all timepoints for dataset 2.**

| **Measure** | **Total Predictions** | **Accuracy** | **Sensitivity** | **Specificity** |
| --- | --- | --- | --- | --- |
| PHQ9 | 111847 | 83% | 50% | 96% |
| GAD7 | 111521 | 77% | 65% | 87% |

**Table G. Timepoints 3 to 10 (when predictions are visible on SilverCloud) for dataset 2.**

| **Measure** | **Total Predictions** | **Accuracy** | **Sensitivity** | **Specificity** |
| --- | --- | --- | --- | --- |
| PHQ9 | 49549 | 88% | 78% | 93% |
| GAD7 | 49407 | 87% | 87% | 90% |

**Table H. PHQ-9 by timepoint for dataset 2 (n=****31149, total RI=8299).**

| **Timepoint** | **Total Predictions** | **RI** | **Correctly Predicted RI** | **No RI** | **Correctly Predicted No-RI** | **Accuracy** | **Sensitivity** | **Specificity** |
| --- | --- | --- | --- | --- | --- | --- | --- | --- |
| 1 | 31149 | 8299 | 0 | 22850 | 22850 | 73.00% | 0.00% | 100.00% |
| 2 | 31149 | 8299 | 3815 | 22850 | 22025 | 83.00% | 46.00% | 96.00% |
| 3 | 22733 | 6942 | 4801 | 15791 | 14817 | 86.00% | 69.00% | 94.00% |
| 4 | 14559 | 4845 | 3983 | 9714 | 9074 | 90.00% | 82.00% | 93.00% |
| 5 | 7500 | 2669 | 2281 | 4831 | 4452 | 90.00% | 85.00% | 92.00% |
| 6 | 3565 | 1314 | 1184 | 2251 | 2051 | 91.00% | 90.00% | 91.00% |
| 7 | 978 | 373 | 325 | 605 | 557 | 90.00% | 87.00% | 92.00% |
| 8 | 194 | 81 | 66 | 113 | 109 | 90.00% | 81.00% | 96.00% |
| 9 | 19 | 8 | 5 | 11 | 10 | 79.00% | 62.00% | 91.00% |
| 10 | 1 | 0 | 0 | 1 | 1 | 100.00% |  | 100.00% |

**Table I. GAD-7 by timepoint for dataset 2 (n=31057, total RI=12026).**

| **Timepoint** | **Total Predictions** | **RI** | **Correctly Predicted RI** | **No RI** | **Correctly Predicted No-RI** |  | **Accuracy** | **Sensitivity** | **Specificity** |
| --- | --- | --- | --- | --- | --- | --- | --- | --- | --- |
| 1 | 31057 | 12026 | 5139 | 19031 | 14224 |  | 62.00% | 43.00% | 75.00% |
| 2 | 31057 | 12026 | 6608 | 19031 | 17691 |  | 78.00% | 55.00% | 93.00% |
| 3 | 22684 | 9775 | 7287 | 12909 | 11793 |  | 84.00% | 75.00% | 91.00% |
| 4 | 14509 | 6586 | 5574 | 7923 | 7242 |  | 88.00% | 85.00% | 91.00% |
| 5 | 7475 | 3562 | 3178 | 3913 | 3486 |  | 89.00% | 89.00% | 89.00% |
| 6 | 3550 | 1706 | 1564 | 1844 | 1654 |  | 91.00% | 92.00% | 90.00% |
| 7 | 975 | 498 | 453 | 477 | 435 |  | 91.00% | 91.00% | 91.00% |
| 8 | 194 | 105 | 94 | 89 | 80 |  | 90.00% | 90.00% | 90.00% |
| 9 | 19 | 11 | 10 | 8 | 8 |  | 95.00% | 91.00% | 100.00% |
| 10 | 1 | 0 | 0 | 1 | 1 |  | 100.00% |  | 100.00% |

## **Dataset 3**

**Region:** UK

**Total Users**: 10627

**Service:** Single health care service

**Programs**: Multiple

**Treatment Mode:** Supported

**Age:** 18+

**Table J. User programs for dataset 3.**

| **Name** | **n** |
| --- | --- |
| 'Space from Depression & Anxiety' | 2927 |
| 'Space from GAD' | 2390 |
| 'Space from Depression' | 2018 |
| 'Space from Stress' | 529 |
| 'Space from Anxiety' | 449 |
| 'Space from Social Anxiety' | 368 |
| 'Space from Panic' | 355 |
| 'Space for Resilience' | 305 |
| 'Space from Health Anxiety' | 294 |
| 'Space from OCD' | 223 |
| 'Space for Perinatal Wellbeing' | 171 |
| 'Space in Chronic Pain from Depression & Anxiety' | 170 |
| 'Space from Phobia' | 163 |
| 'Space for Sleep' | 151 |
| 'Space in Lung Conditions from Depression & Anxiety' | 29 |
| 'Space for Diabetes Wellbeing' | 26 |
| 'Space from Money Worries' | 25 |
| 'Space in Diabetes from Depression & Anxiety' | 19 |

**Table K. Average for all timepoints for dataset 3.**

| **Measure** | **Total Predictions** | **Accuracy** | **Sensitivity** | **Specificity** |
| --- | --- | --- | --- | --- |
| PHQ9 | 42850 | 82% | 51% | 96% |
| GAD7 | 42772 | 77% | 64% | 87% |

**Table L. Timepoints 3 to 10 (when predictions are visible on SilverCloud) for dataset 3.**

| **Measure** | **Total Predictions** | **Accuracy** | **Sensitivity** | **Specificity** |
| --- | --- | --- | --- | --- |
| PHQ9 | 21600 | 88% | 76% | 94% |
| GAD7 | 21552 | 85% | 79% | 90% |

**Table M. PHQ-9 by timepoint for dataset 3(n=****10625, total RI=2986).**

| **Timepoint** | **Total Predictions** | **RI** | **Correctly Predicted RI** | **No RI** | **Correctly Predicted No-RI** | **Accuracy** | **Sensitivity** | **Specificity** |
| --- | --- | --- | --- | --- | --- | --- | --- | --- |
| 1 | 10625 | 2986 | 0 | 7639 | 7639 | 72.00% | 0.00% | 100.00% |
| 2 | 10625 | 2986 | 1275 | 7639 | 7400 | 82.00% | 43.00% | 97.00% |
| 3 | 8442 | 2606 | 1694 | 5836 | 5532 | 86.00% | 65.00% | 95.00% |
| 4 | 6161 | 2027 | 1599 | 4134 | 3893 | 89.00% | 79.00% | 94.00% |
| 5 | 3810 | 1299 | 1084 | 2511 | 2340 | 90.00% | 83.00% | 93.00% |
| 6 | 1983 | 722 | 623 | 1261 | 1167 | 90.00% | 86.00% | 93.00% |
| 7 | 875 | 330 | 287 | 545 | 502 | 90.00% | 87.00% | 92.00% |
| 8 | 283 | 105 | 93 | 178 | 161 | 90.00% | 89.00% | 90.00% |
| 9 | 45 | 15 | 13 | 30 | 29 | 93.00% | 87.00% | 97.00% |
| 10 | 1 | 1 | 1 | 0 | 0 | 100.00% | 100.00% |  |

**Table N. GAD-7 by timepoint for dataset 3 (n=4456, total RI=1905).**

| **Timepoint** | **Total Predictions** | **RI** | **Correctly Predicted RI** | **No RI** | **Correctly Predicted No-RI** | **Accuracy** | **Sensitivity** | **Specificity** |
| --- | --- | --- | --- | --- | --- | --- | --- | --- |
| 1 | 10610 | 4456 | 1905 | 6154 | 4686 | 62.00% | 43.00% | 76.00% |
| 2 | 10610 | 4456 | 2249 | 6154 | 5730 | 75.00% | 50.00% | 93.00% |
| 3 | 8426 | 3824 | 2630 | 4602 | 4185 | 81.00% | 69.00% | 91.00% |
| 4 | 6143 | 2948 | 2382 | 3195 | 2909 | 86.00% | 81.00% | 91.00% |
| 5 | 3803 | 1898 | 1665 | 1905 | 1695 | 88.00% | 88.00% | 89.00% |
| 6 | 1977 | 1044 | 941 | 933 | 836 | 90.00% | 90.00% | 90.00% |
| 7 | 875 | 483 | 445 | 392 | 355 | 91.00% | 92.00% | 91.00% |
| 8 | 282 | 155 | 141 | 127 | 117 | 91.00% | 91.00% | 92.00% |
| 9 | 45 | 29 | 25 | 16 | 16 | 91.00% | 86.00% | 100.00% |
| 10 | 1 | 1 | 1 | 0 | 0 | 100.00% | 100.00% |  |

## **Dataset 4**

**Region:** UK

**Total Users**: 82

**Service:** Single health care service

**Program:** Space from Depression & Anxiety

**Treatment Mode:** Supported

**Age:** 18+

The 82 users in this dataset are a subset of users from a UK based service who participated in a pragmatic RCT to test the effectiveness of SilverCloud Health compared to a waitlist control group and gave consent for the collection and dissemination of demographic and clinical information.

**Table O. PHQ-9 gender comparison for dataset 4.**

| **Gender** | **Total Predictions** | **Accuracy** | **Sensitivity** | **Specificity** |
| --- | --- | --- | --- | --- |
| Female | 186 | 87% | 49% | 97% |
| Male | 112 | 87% | 47% | 98% |

**Table P. GAD-7 gender comparison for dataset 4.**

| **Gender** | **Total Predictions** | **Accuracy** | **Sensitivity** | **Specificity** |
| --- | --- | --- | --- | --- |
| Female | 186 | 78% | 59% | 88% |
| Male | 112 | 85% | 63% | 94% |

**Table Q. PHQ-9 ethnicity comparison for dataset 4.**

| **Ethnicity** | **Total Predictions** | **Accuracy** | **Sensitivity** | **Specificity** |
| --- | --- | --- | --- | --- |
| Non-White British | 36 | 83% | 35% | 95% |
| White British | 262 | 87% | 51% | 98% |

**Table R. GAD-7 ethnicity comparison for dataset 4.**

| **Ethnicity** | **Total Predictions** | **Accuracy** | **Sensitivity** | **Specificity** |
| --- | --- | --- | --- | --- |
| Non-White British | 36 | 78% | 58% | 84% |
| White British | 262 | 81% | 60% | 92% |

**Table S. PHQ-9 age group comparison for dataset 4 (median age=29).**

| **Age Group** | **Total Predictions** | **Accuracy** | **Sensitivity** | **Specificity** |
| --- | --- | --- | --- | --- |
| < 30 | 155 | 92% | 55% | 98% |
| >=30 | 146 | 82% | 68% | 90% |

**Table T. GAD-7 age group comparison for dataset 4 (median age=29).**

| **Age Group** | **Total Predictions** | **Accuracy** | **Sensitivity** | **Specificity** |
| --- | --- | --- | --- | --- |
| < 30 | 155 | 83% | 66% | 89% |
| >=30 | 146 | 83% | 62% | 92% |

1. <https://uk.silvercloudhealth.com/help/privacy/> [↑](#footnote-ref-2)
