# Supplementary material for: Deep learning for the prediction of clinical outcomes in internet-delivered CBT for depression and anxiety
Source: PLoS One. 2023 Nov 27;18(11):e0272685. doi: 10.1371/journal.pone.0272685 (PMC10681250; doi:10.1371/journal.pone.0272685)
Supplement: S1 Appendix — Fig 1. User distribution and reliable improvement over time. Top figure: Distribution of time from enrolment to nth review; bottom figure: Counts of users at each review period. Fig 2. Kaplan Meier curves modelling reliable improvement events in PHQ-9 and GAD-7 for clients with different baseline severity. Survival probability corresponds to the probability that sustained reliable improvement is not achieved by a given timestep in the treatment program. A steeper drop in survival probability can be interpreted as higher rate of reliable improvement in that subpopulation. (DOCX) [file pone.0272685.s005.docx]

# Appendix C: User Distribution and Reliable Improvement over time


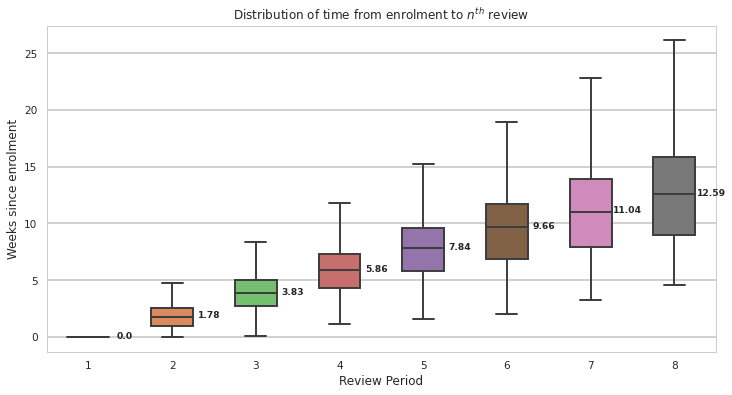

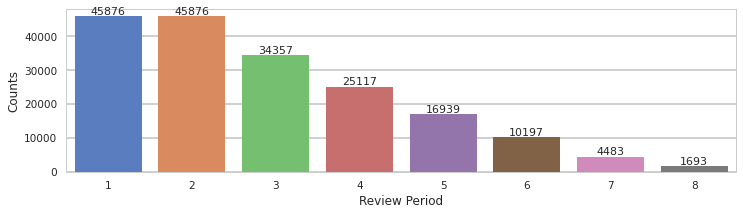


**Figure C1:** (a) Distribution of time from enrolment to nth review, (b) Counts of users at each review period


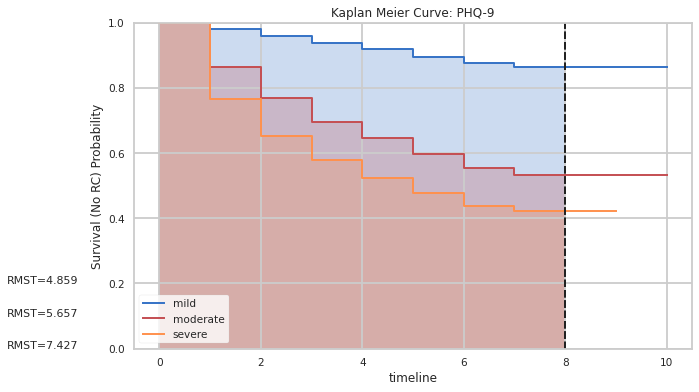

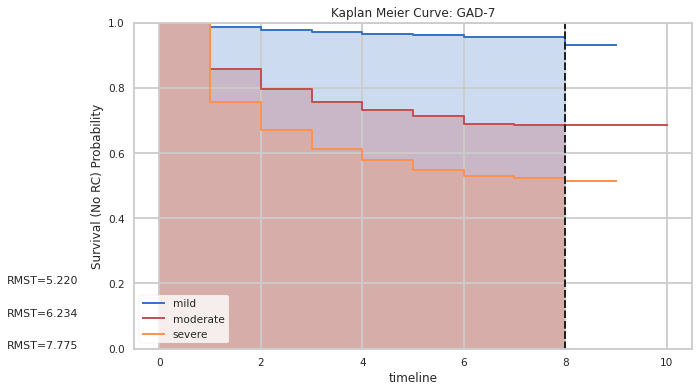


**Figure C2:** Kaplan Meier curves modelling reliable improvement events in (a) PHQ-9, (b) GAD-7 for clients with different baseline severity. Survival probability corresponds to the probability that sustained reliable improvement is not achieved by a given timestep in the treatment program. A steeper drop in survival probability can be interpreted as higher rate of reliable improvement in that subpopulation.
